# Nanoparticles and cytokine response

**DOI:** 10.3389/fbioe.2023.1243651

**Published:** 2023-08-28

**Authors:** Mohammad Nasrullah, Daniel Nisakar Meenakshi Sundaram, Jillian Claerhout, Khanh Ha, Erkan Demirkaya, Hasan Uludag

**Affiliations:** ^1^ Faculty of Pharmacy and Pharmaceutical Sciences, University of Alberta, Edmonton, AB, Canada; ^2^ Department of Chemical and Materials Engineering, Faculty of Engineering, University of Alberta, Edmonton, AB, Canada; ^3^ Department of Paediatrics, Schulich School of Medicine & Dentistry, Western University, London, ON, Canada; ^4^ Department of Biomedical Engineering, Faculty of Medicine and Dentistry, University of Alberta, Edmonton, AB, Canada

**Keywords:** cytokine response, nanoparticle, inflammatory response, non-viral delivery, biocompatibility

## Abstract

Synthetic nanoparticles (NPs) are non-viral equivalents of viral gene delivery systems that are actively explored to deliver a spectrum of nucleic acids for diverse range of therapies. The success of the nanoparticulate delivery systems, in the form of efficacy and safety, depends on various factors related to the physicochemical features of the NPs, as well as their ability to remain “stealth” in the host environment. The initial cytokine response upon exposure to nucleic acid bearing NPs is a critical component of the host response and, unless desired, should be minimized to prevent the unintended consequences of NP administration. In this review article, we will summarize the most recent literature on cytokine responses to nanoparticulate delivery systems and identify the main factors affecting this response. The NP features responsible for eliciting the cytokine response are articulated along with other factors related to the mode of therapeutic administration. For diseases arising from altered cytokine pathophysiology, attempts to silence the individual components of cytokine response are summarized in the context of different diseases, and the roles of NP features on this respect are presented. We finish with the authors’ perspective on the possibility of engineering NP systems with controlled cytokine responses. This review is intended to sensitize the reader with important issues related to cytokine elicitation of non-viral NPs and the means of controlling them to design improved interventions in the clinical setting.

## 1 Introduction

Nucleic acid-based therapies utilize a range of endogenous and altered nucleic acids derived from DNA and RNA to target specific genes and modify their expression or activity, which lead to therapeutic benefits. A handful of nucleic acid therapies have gained entry into the clinical practice while a large range of nucleic acids are now undergoing clinical testing for regulatory approval. They offer several advantages over conventional drugs (i.e., small organic molecules) and provide activities that cannot be matched by such drugs. They can be designed to be highly specific, avoiding off-target effects that can occur with the current drugs. Rather than simply suppressing the symptoms, these therapies have the potential to treat diseases at the genetic level by altering the root-cause of the disease with long-lasting effects, allowing targeted and precise treatments (Kulkarni et al., 2021). Nucleic acid-based therapies derived from plasmid DNA (pDNA), messenger RNA (mRNA), small interfering RNA (siRNA), and antisense oligonucleotides (ASOs), have shown promise in treating a range of diseases, including those that are currently considered “incurable”. The siRNA-based drug Vutrisiran, to treat the rare genetic disease polyneuropathy of hereditary transthyretin-mediated amyloidosis (Mullard, 2022) and the mRNA-based COVID-19 vaccines (Watson et al., 2022) are leading examples of nucleic acid therapies.

With the help of recent technological advancements, nucleic acid therapies have become progressively feasible in terms of both production and accessibility, hence, they are explored in clinics for many indications. A critical challenge in exploring new indications lies in the efficiency of the delivery system (Uludag et al., 2019). Administering nucleic acids without a carrier or delivery system is not desirable since they cannot enter cells on their own (at least without significant chemical modifications). These nucleic acids are also prone to undergo rapid degradation due to systemic nucleases, and elicit immune responses due to similarities with the genetic make-up of foreign entities. Nanoparticles (NPs), i.e., delivery vesicles structured at the nm-scale, have emerged as the most promising candidates for delivering nucleic acid-based drugs to overcome such challenges. Their success depends on their ability to efficiently reach the target cells and release their payload without inducing significant toxicity. The NPs are non-viral in nature, but they could still interact with the immune system and subsequently trigger immune responses that can lead to adverse effects. Cytokine response is considered as one of the key immune responses to the NPs (Zolnik et al., 2010) and it is imperative for NPs to remain in “stealth” model when it comes to cytokine response upon administration in a host.

Cytokines are a class of proteins with essential functions in immunity, and other physiological processes such as cellular proliferation, differentiation, apoptosis and inflammatory reactions. They are produced and secreted by various cells in the body, including immune cells and endothelial cells in response to a variety of physiological events and pathological processes. They act as chemical messengers, which bind to specific receptors on the surface of target cells, triggering a range of downstream signaling pathways, and facilitating communications between cells of the immune system, as well as other cells in the body. Cytokines can be broadly classified into several categories, including growth factors, interleukins (ILs), interferons (IFNs), tumor necrosis factors (TNFs), and chemokines (Deckers et al., 2023). Abnormal cytokines levels can trigger a pro-inflammatory or anti-inflammatory response and are associated with a wide range of diseases. Excessive secretion of certain cytokines has been linked to the development of autoimmune diseases such as rheumatoid arthritis, lupus, and multiple sclerosis, in which the body’s immune system erroneously targets healthy cells and tissues (Moudgil and Choubey, 2011). Sepsis is induced by overproduction of cytokines in response to foreign invaders and can lead to death if uncontrolled. Certain cytokines, such as IL-15 and M-CSF may promote tumorigenesis, whereas others, such as IFN-γ, may inhibit it (Dranoff, 2004). Cytokines can typically display multiple roles, such as TNF-α and IFN-γ, which can either promote tumor growth or suppress it (Wang and Lin, 2008). Cytokine alterations are also the root-cause of certain chronic inflammatory disorders such as ulcerative colitis, Crohn’s disease, and inflammatory bowel disease. Depression and other mood disorders have been linked to elevated levels of pro-inflammatory cytokines (Raison et al., 2006).

When NPs enter the body bearing nucleic acids, they can be recognized by the immune system as foreign invaders like viruses, which can activate monocytes, macrophages, and dendritic cells to release defensive cytokines. Several factors related to the physicochemical features of the NPs, the nucleic acid cargo, administration regimen (location, dose, etc.) and patients’ genetic profile can influence the nature and the intensity of the cytokine response (Gonçalves et al., 2020; Bila et al., 2021; Gu et al., 2021). In extreme cases, a cytokine storm, also known as cytokine release storm (Fajgenbaum and June, 2020) may be triggered. On the other hand, a controlled cytokine response with modulation of desired cytokines, can be beneficial by enhancing immune responses against tumors or pathogens, which leads to better therapeutic outcomes. Hence, understanding the cytokine response to NPs is essential to investigate the safety and efficacy of nucleic acid-based drugs.

In this report, we cover the latest developments in the cytokine response to NP-based non-viral delivery systems. We provide a brief insight into the premises behind different NP formulations, highlight their technological features that make them promising for clinical translation and survey the information on their cytokine response. We discuss ideas that may pave the way to interfere specifically with abnormal cytokine physiology and develop improved formulations with minimal adverse effects.

## 2 Nanoparticle-based nucleic acid delivery systems

**FIGURE 1:**
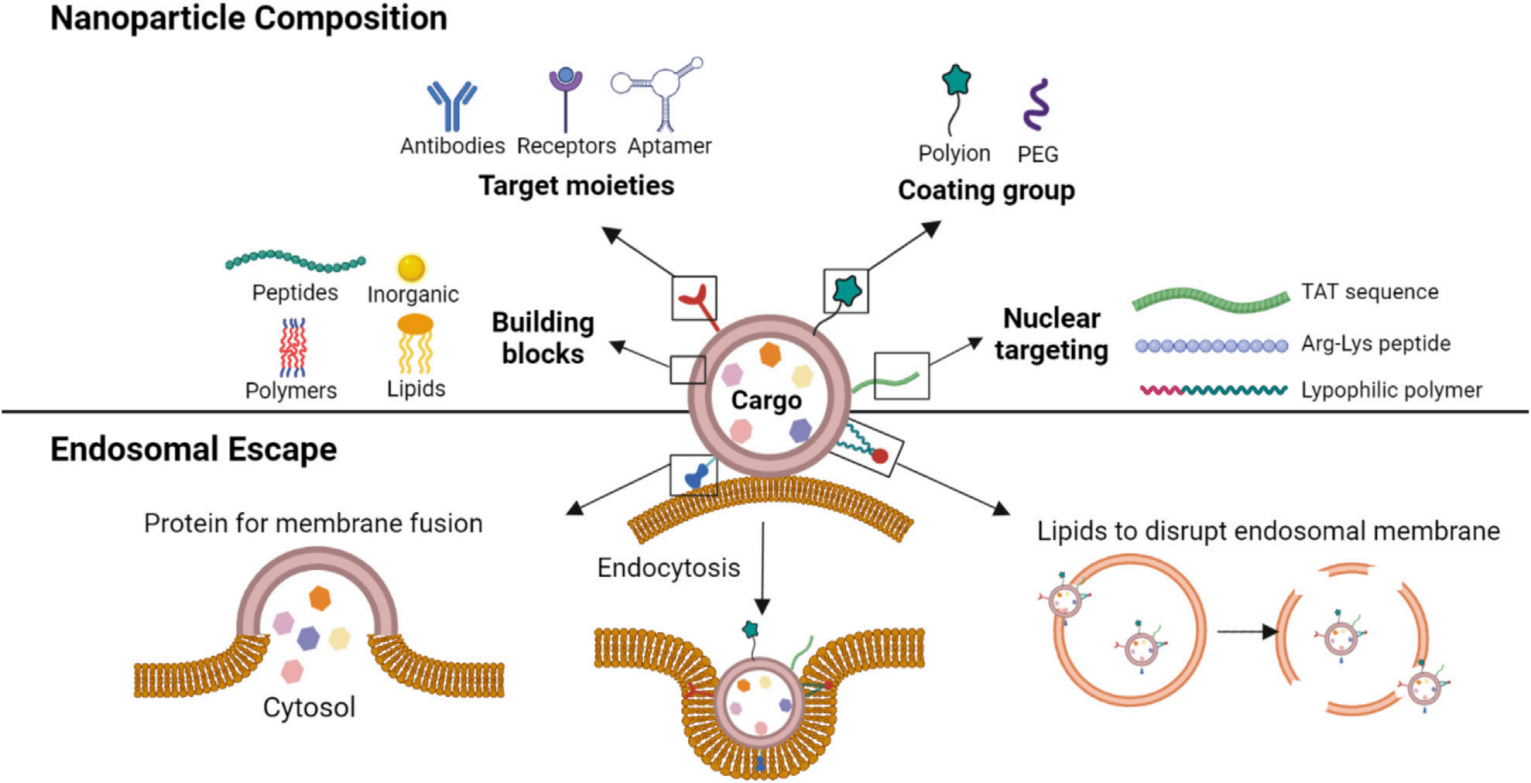
A schematic representation of various constituents of a NP. The NPs are constructed from several types of building blocks with, if necessary, targeting moieties (for active targeting to a cell/site), coating groups (to reduce opsonization and/or non-specific interactions) and nuclear localization signals (for cell nucleus targeting). The lipid constituents of the NP could further facilitate membrane fusion and/or destabilization of endosomal membranes to prevent endosomal entrapment of the cargo.

A diverse range of materials has been explored as the foundation for NP fabrication in delivery of nucleic acids (Figure 1). These include peptide/protein derived natural or synthetic biomolecules, lipid-based compounds (natural and synthetic), polymeric and dendrimeric materials, inorganic frameworks (Paunovska et al., 2022), and more recently exosomes (El-Andaloussi et al., 2012). Cationic biomaterials are used to take advantage of the anionic nature of the nucleic acids to form ionic complexes for NP formation and/or entrapment, while the neutral biomaterials have been used for simple entrapment or encapsulation of nucleic acids. Some NPs are derived from a single (homogenous) component, which allows a simple fabrication process but may limit functional features of the NPs. On the other hand, the leading NP formulation, lipid NPs (LNPs) that formed the basis of the recent SARS-CoV-2 vaccines, are formed from a cocktail of lipids: a neutral lipid (e.g., distearoylphosphatidylcholine, DSPC) for nucleic acid entrapment, cholesterol for structure integrity, PEGylated lipid (DSPE-PEG) to create a hydrophilic, non-opsonizing surface, and an ionizable lipid (e.g., DLin MC3-DMA) for membrane fusion (Verbeke et al., 2019; Wang et al., 2023). Having multiple components enhances the functionality of NPs but may require specialized fabrication techniques not readily amenable for scale-up. Advanced polymers may incorporate some of these features into a single component, such as cationic and PEG moieties, lipidic side-groups and bioactive peptides capable of controlling intracellular trafficking (Suk et al., 2016), so that NPs formulations can be conveniently prepared as long as the single component polymer is fabricated in a reproducible manner.

Irrespective of the building block, the NPs can facilitate nucleic acid delivery into cells through various uptake mechanisms, including cell membrane penetration and receptor-mediated internalization, and further binding to nucleic acids to protect them from degradation in the extracellular environment. Where necessary, cell/tissue/organ trophy can be built into the NPs by selective binding ligands that include biosynthetic antibodies against desired molecules (e.g., Her2), particular receptor ligands (e.g., folate) (Xu et al., 2017) or aptamers, to name a few. Such targeted systems aim to concentrate the nucleic acids at sites of action, and are expected to elicit minimal host response that can prevent the NPs from functioning.

Since endosomal membranes pose a significant barrier to the transport and release of the nucleic acids, NPs with endosomal escape mechanisms are preferred in the design of the carriers. Several strategies have been utilized to this end; if possible, endosomal entrapment could be avoided by using “fusogenic” NPs that can fuse with the cell membrane and release their cargo into the cytoplasm (Chen et al., 2013; Joubert et al., 2023 ). Lipid NPs (LNPs), in particular, can fuse with cell membranes to release their cargo into the cytoplasm directly. However, all NPs are likely to undergo some extent of endosomal uptake and the primary approach to overcome this barrier is to incorporate specific signals that facilitate endosomal escape such as the fusogenic peptides (Samec et al., 2022; Joubert et al., 2023), or lipids that disrupt the endosomal membrane order (Lam et al., 2023). Alternatively, some polymers can display “proton sponge” effect, in which the cationic moieties absorbs H^+^ and produces osmotic swelling of the endosomes, resulting in rupture and discharge of the nucleic acids into the cytoplasm (Pei and Buyanova, 2019). The release of the cargo from endosomes might be sufficient for nucleic acids that function in cytoplasm, for example, mRNA and non-coding RNAs such as short interfering RNA (siRNA), but additional measures might be needed to enhance the nuclear uptake of the nucleic acid. The nuclear uptake can be facilitated with lysine-arginine rich synthetic polypeptides (Li et al., 2022), viral-derived TAT peptides, ionizable lipids or lipophilic polymers whose mechanism of nuclear uptake remains to be elucidated can function along the same lines and increase nucleic acid uptake into cells.

## 3 Cytokine response to nanoparticles

**FIGURE 2:**
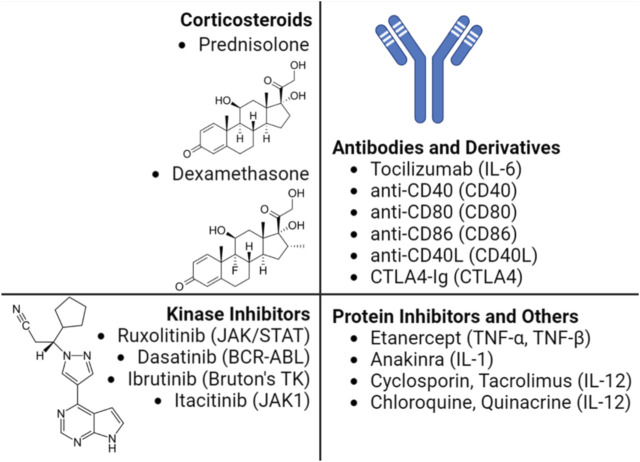
Main pharmacological agents employed to control cytokine storm in a clinical setting. The mediators targeted by the pharmacological agent is shown in parenthesis. The immunosuppressive properties of the corticosteroids are attributed to their ability to bind glucocorticoid receptors to block various signaling pathways affecting cytokine production in immune cells. The humanized antibody Tocilizumab predominantly used in treating rheumatoid arthritis also received approval to address cytokine release syndrome (CRS) following a CAR-T therapy based on AAV (Le et al., 2018). The kinase inhibitors with indicated target enzyme displayed immuno suppressive properties, including suppression of CRS, some of which are being investigated in T-cell therapies (Fraietta et al., 2016; Huarte et al., 2020; Xiang et al., 2022). Etanercept and Anakinra was successfully used to address CRS in patients without altering the therapeutic effect of CAR-T therapy in treatment of multiple myeloma (Zhang L et al., 2021) and B-cell lymphoma (Strati et al., 2020), respectively. Other molecules such as cyclosporin and tacrolimus, which inhibit the phosphatase calcineurin, can downregulate the IL-2 cytokine levels (Chu and Ng, 2021).

The ability of a host to mount a significant cytokine release in response to administered nucleic acids with various delivery platforms has been appreciated. The most common clinical approach to circumvent immune response is through the administration of immunosuppressive agents to block immune cell interaction (Zhang C et al., 2021). Figure 2 provides a summary of various pharmacological approaches to interfere with the cytokine response in a clinical setting, not necessarily tailored for the NP systems. Some of the utilized agents are broad acting such as the use of corticosteroids, while others are quite specific such as the use of Anakinra^TM^ to inhibit IL-1β activity. Early clinical studies using viral delivery of gene medicines indicated the cytokine response to be significant; following an adenovirus-based infusion protocol into the right hepatic artery for the treatment of ornithine transcarbamylase deficiency, 1 patient (out of 19) exhibited peak levels of IL-6 (∼4,500 pg/mL) at 8 h post injection that remained high until the passing of the patient (Chatenoud et al., 1989; Raper et al., 2003). Although one other patient also exhibited high IL-6 level, it subsequently dropped helping in complete recovery. Another study with an adenovirus vector in 2009 carrying human hepatocyte growth factor (Ad-HGF) proved successful involving 21 patients with severe coronary artery disease. While IL-4 levels remained unaffected by the treatment, IL-10 was upregulated in the first 24 h, but later decreasing to control levels (Yang et al., 2009). To improve the safety, a non-viral approach was employed for cystic fibrosis correction with lipid (DOPE:DMPE–PEG_5000_)/DNA complexes in an aerosolized form; 4 out of 8 patients experienced fever, muscle, and joint aches along with myalgias and arthralgia in some of them. Serum IL-6 levels were elevated in all patients ranging between ∼10 and ∼50 pg/mL although no causality was reported, while IL-1, IL-8, TNFα and IFN-γ levels were unaltered (Ruiz et al., 2001). A polymeric NP, CALAA-01 consisting of i) a cationic cyclodextrin, ii) a hydrophilic stabilizer adamantane-PEG and iii) a targeting ligand for human transferrin receptor, was used to carry an siRNA against an anti-cancer target, ribonucleotide reductase M2 subunit (RRM2). The serum cytokine studies in primates recorded an upregulation of IL-6, IL-12 and IFN-γ levels which correlated with patient clinical trials (*n* = 24). The cytokine levels peaked mostly between 2 and 6 h post injection with IL-6 and IL-10 reaching ∼600 pg/mL, TNFα ∼200 pg/mL and IFN-γ ∼50 pg/mL, but the levels dropped back to normal/baseline in 24 h. It was evident that the polymeric CALAA-01 induced an inflammatory response but was tolerated to some extent by the patients (Zuckerman et al., 2014).

Recent studies in preclinical models have provided alternative, non-pharmacological approaches to control the cytokine response to non-viral carriers. Dose is an important contributing factor as higher doses correlate with higher cytokine induction in several models. Although no major cytokine upregulation was reported with cell penetrating peptides (CPPs), a slight upregulation could be seen in TNF-α levels between 1 and 5 mg/kg doses of CPPs TP10, stearyl-(RxR)_4_, PF3 and PF4 *in vivo* (Suhorutsenko et al., 2011). Similarly, the levels of IL-1β/-6/-8 secretion from keratinocytes/fibroblasts increased with increase in the dose of PAMAM *in vitro* (Czarnomysy et al., 2019) as well as cytokine secretion (MIP-2, TNF-α and IL-6) from macrophage cells to G4-G6 PAMAMs (Naha et al., 2010). Among 7 different lipid-modified LMW bPEI, one polymer showed increased IL-6/TNF-α secretion at higher polymer dose (∼20 pg/mL vs. < 5 pg/mL) (Meenakshi Sundaram et al., 2022).

Increase in pDNA dose from 30 μg to 80 µg increased the serum TNF-α levels from ∼5 to ∼40 pg/mL (Kawakami et al., 2006). Similar observations were reported with oligodeoxynucleotides having CpG motifs as the levels of IL-6 and IFN-α increased from ∼500 to ∼1,250 pg/mL and ∼80–∼180 pg/mL, respectively, with increase in the concentration of the oligodeoxynucleotides delivered by a 1.8 kDa PEI (Cheng et al., 2018). Such a dose-dependent increase in cytokine levels was also seen with CALAA-01 NPs, as patients treated with 10 mg/m^2^ (dose of siRNA) displayed negligible cytokine response, while with 20–30 mg/m^2^ dose, there was a maximum increase of 200-fold for IL-6, 100-fold for IL-10, 15-fold for TNF-α and 20-fold for IFN-γ (Brockstedt et al., 1999; Zuckerman et al., 2014). Use of a low dose should be a key consideration irrespective of the therapy and should be implemented even at the earliest stages of discovery and validation studies in *in vitro* and *in vivo* bioassays.

**FIGURE 3:**
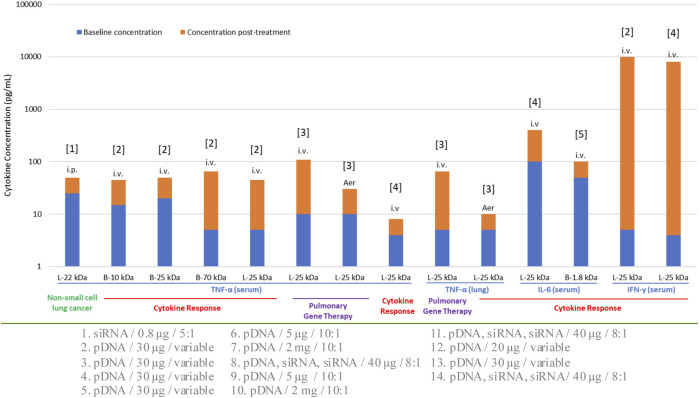
Cytokine response to various formulations of PEI in preclinical animal models. The specific cytokine investigated, the administration route (IV, IP, AER; intravenous, intraperitoneal and aerosolized, respectively) and the normal/disease model employed are indicated for each study. L: Linear PEI, B: Branched PEI. The numbers on the horizontal axis indicate the molecular weight (kDa) of the PEI used. The nature of the nucleic acid cargo, the dose administered and the N:P ratio employed in the formulations were variable; this information is provided below the graph and numbered according to the bar number from left to right. References for the studies are: [1]: Zhang et al., 2013, [2]: Kawakami et al., 2006, [3]: Gautam et al., 2001, [4]: Bonnet et al., 2008, [5]: Cheng et al., 2018.

Selection of administration route is normally dependent on the type of therapeutic intervention in addition to the anatomical site being targeted. Local routes would be more beneficial to confine the NPs, thereby avoiding unwanted systemic side effects. Commonly utilized IV route has the advantage of targeting systemic diseases as it can reach various organs. The influence of administration route on immune response was recognized early when using recombinant adeno-associated viral vectors (AAV) in C57BL/6 mice for ovalbumin delivery. The immune response following IM injection showed the lowest response through the development of cytotoxic T lymphocytes (CTL) (Brockstedt DG et al., 1999), while contrasting effects were observed in other studies where IM route triggered a high immune response compared to other routes of administration. Additional studies on the difference in immune responses upon different routes of administration (oral, IV and IM) have been reported with AAV through CTL (Sun et al., 2003; Shirley et al., 2020). Relatively fewer studies could be found with non-viral gene delivery systems. Routes such as IM, intradermal (ID), intralymphatic (ILy), and SC were compared in BALB/c mice with a model antigen (ovalbumin). All systems showed an antibody response with ILy administration. The ID and IM routes induced a moderate response, whereas the SC route did not elicit any response with T-helper (Th) cells being a major player in this study. Re-stimulation of the mice splenocytes (*in vitro*) was performed to better understand the cellular response based on IFN-γ, IL-4 and IL-10 cytokine secretion. With liposomes, the ILy route showed an elevated IFN-γ secretion compared to the SC, ID and IM routes. The chitosan and PLGA NPs did not show any major differences between the route of administrations and IFN-γ/IL-10 induction, while the route of administration was important for chitosan NPs for the IL-4 secretion (IM < SC < ID < ILy) (Mohanan et al., 2010). Although this was a vaccination study, the findings could be extrapolated to gain an insight on the immunogenicity of non-viral NPs. With PEI-based vectors, several animal studies were reported for nucleic acid NPs derived from this carrier (Figure 3) and different routes did not appear to make a significant difference in the elicited response in this case. Within this limited data set, i) some cytokines (IFN-γ) appeared to be more stimulated with this particular carrier, and ii) the cytokine response appeared to be similar in normal vs. disease models. Different types of NPs other than LNPs and polymeric systems also found to contribute to cytokine responses (Table 1).

**TABLE 1:** Cytokines responses by NPs with/without cargos other LNPs and polymeric systems.

Type of NPs	Composition of NP (−/+) cargo	Cytokine and functional response
Carbon nanotube (CN)	Multi-wall CN with a mixture of 5.6 wt% ferrocene in toluene	Induces pro-inflammatory cytokines (Boyles et al., 2015)
	Single-wall CN with amine-functionalized group	Induces TNFα and IL-1β (Gao et al., 2021a)
Graphene oxide (GO)	GO from Asbury Mills 3061 grade graphite	GO with larger lateral size enhances the production of IL-6, TNFα and IL-1β (Ma et al., 2015)
	Vanillin-functionalized NP from a mixture of vanillin with GO	Releases significantly higher level of IL1-β, TNF-α, GM-CSF, IL-6, IL-8 (Gurunathan et al., 2019)
Dendrimers	Non-modified native PAMAM dendrimer	Triggers secretion of TNFα and IL-1β (Gao et al., 2021b)
Magnetic nanoparticle (MNP)	MNP prepared by dissolving iron(III) acetylacetonate, 1,2-hexadecanediol, oleic acid, and oleylamine in benzyl ether	Suppresses chronic inflammatory response and activates acute inflammatory response (Zhu et al., 2019)
Gold NP (GNP)	*Ephedra sinica* Stapf extract-capped GNP	Helped *Ephedra sinica* Stapf to silence upstream signaling pathways of pro-inflammatory mediators and cytokines (Park et al., 2019)
Gold NP (GNP)	*Hypericum perforatum* extract-capped GNP	Helped *Hypericum perforatum* to downregulate pro-inflammatory cytokines (IFN-γ, IL-17A and IL-6) and upregulate anti-inflammatory cytokines (TGF-β, IL-10 and IL-4) (Mahmoudi et al., 2022)
Silica NP (SNP)	Mesoporous SNP	Induces the expression pro-inflammatory cytokine genes (IL-1, IL-8 and TNF-α) (Ghasemi et al., 2022)
SNP		Induces pro-inflammatory cytokines CXCL8 and IL-6 (Låg et al., 2018)
Calcium phosphate NP (CaPNP)	CapNP by incubating BSA and CaCl2 in Dulbecco’s Modified Eagle Medium	Facilitated delivery of TNF-stimulated gene 6 knocked mesenchymal stem cells into liver and silences cytokines (Wang et al., 2020)
Exosome	Obtained from adipose-derived mesenchymal stem cells	Induces higher expression of anti-inflammatory cytokines (Heo et al., 2020)

**FIGURE 4:**
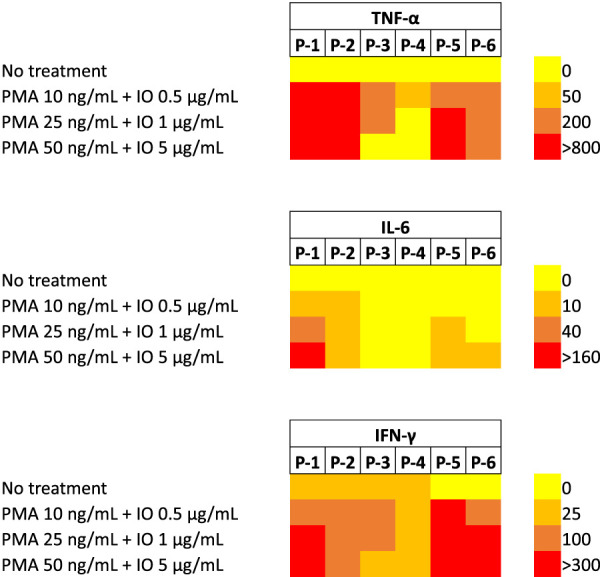
Secretion of inflammatory cytokines (TNF-α, IL-6 and IFN-γ) from PBMCs obtained from 6 separate, healthy donors in culture. The cells were treated with PMA/IO combinations at indicated concentrations and cytokine secretion was determined 3 days afterwards (by specific ELISAs). Untreated cells served as the reference. Note that some PBMCs were unresponsive to the stimulation (especially in IL-6 and TNF-α secretion) while other PBMCs were hyper-sensitive to the stimulation. Significant donor-to-donor variation was evident among the PBMCs when it comes to cytokine secretion upon the stimulation (Meenakshi Sundaram et al., 2022).

With viral delivery systems, pre-exposure to viruses could explain the stronger cytokines responses in some individuals, but this should not be an issue with NPs. In Phase Ia/Ib studies with CALAA-01, 19 (M) Phase Ia patients had good tolerance to dose escalation, while 3 (F) out of 5 (F) Phase Ib patients experienced dose-limiting toxic events (DLTs) along with grade 3 toxicities. Some patients exhibited slight cytokine response mediated by Th2 cells. (Zuckerman et al., 2014). Substantial variation in cytokine induction was observed among 307 participants (children) towards virus and bacterial infection with differences as high as 1000-fold. Twenty-eight separate cytokines were evaluated against 15 different stimuli including various pathogens using peripheral blood mononuclear cells (PBMC) isolated from 307 participants. The reason(s) for the difference could be due to i) different receptors being recognized among the participants, ii) failure to determine the specific cell type present in PBMCs before undertaking the study, iii) use of PBMCs stored in liquid nitrogen and iv) the difference in the time of sample collection along with genetic differences among the cells (Lin et al., 2022). We also noted such differences in *vitro* studies performed with PBMC obtained from otherwise healthy individuals (i.e., blood donors) (Figure 4); only 3 out of 6 donor PBMC exhibited very high levels with the “stimulatory” treatment of PMA/IO (phorbol 12-myristate 13-acetate/ionomycin).

## 4 Management of cytokine response: possibility of precision intervention

**FIGURE 5:**
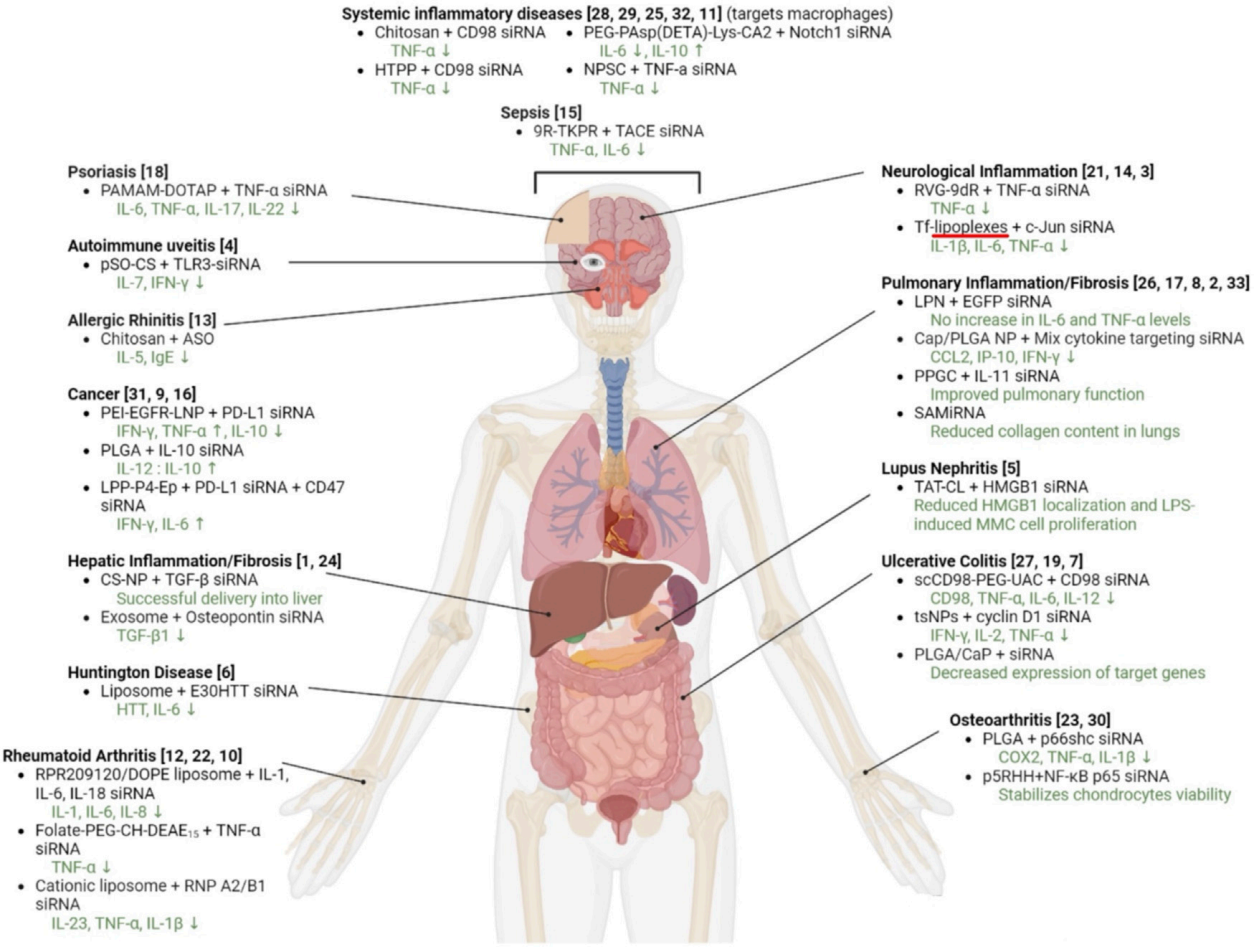
Attempts to implement RNAi in various disease models involving cytokine mediators. The specific siRNA target and the delivery system used are indicated. The references to the specific study are numbered as follows: 1: (Azzam, El Safy, et al., 2020). 2: (Bai et al., 2022). 3: (Cardoso et al., 2010). 4: (Chen et al., 2013). 5: (Diao et al., 2019). 6: (Fihurka et al., 2022). 7: (Frede et al., 2016). 8: (Frede et al., 2017. 9: (Heo et al., 2015). 10: (Herman et al., 2015). 11: (Jiang et al., 2018). 12: (Khoury et al., 2008). 13: (Kim and Kim, 2007). 14: (Kim et al., 2010). 15: (Lee et al., 2021). 16: (Lian et al., 2019). 17: (Okuda et al., 2018). 18: (Pandi et al., 2018). 19: (Peer et al., 2008). 20: (Rudd et al., 2020). 21: (Kim et al., 2011). 22: (Shi et al., 2018). 23: (Shin et al., 2020). 24: (Tang et al., 2022). 25: (Wu et al., 2018). 26: (Wu et al., 2021). 27: (Xiao et al., 2014). 28: (Xiao et al., 2016). 29: (Xiao et al., 2017). 30: (Yan et al., 2019). 31: (Yang et al., 2022). 32: (Jiang et al., 2018). 33: (Yoon et al., 2016).

Novel approaches are being explored to formulate minimally immunogenic NP carriers and for therapeutic purpose in diseases involving cytokine dis-regulation. The collective effort is paving the way for a personalized approach to cytokine therapy whereby very specific interventions, mainly based on RNA Interference (RNAi), are beginning to be employed to control the upregulation of specific cytokines. Short interfering RNA (siRNA), as the pharmacological mediator of RNAi, has gained approval for several clinical indications now (Table 2). Figure 5 summarizes various RNAi approaches to target cytokine mediators in different diseases. The versatility of the RNAi approach through the design of siRNA from a core blueprint is allowing rapid adaptation of this technology for diverse diseases, made possible by conveniently targeting different cytokine mediators suspected to be involved in each particular disease.

**TABLE 2:** Clinically approved siRNAs, their formulation details and administration doses.

Name	Formulation	Administration dose
ONPATTRO® (patisiran) lipid complex injection, for intravenous use	Sequence: RNA: auggaauacu cuugguuact t	IV of 0.3 mg/kg every 3 weeks for patients <100 kg; 30 mg/kg every 3 weeks for patients >100 kg
	complex with RNA: guaaccaaga guauuccaut t	
GIVLAARI® (givosiran) injection, for subcutaneous use	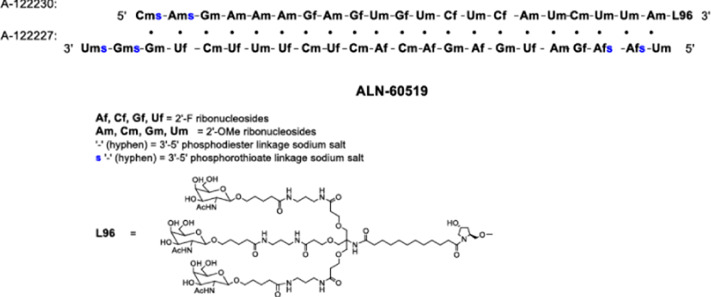	2.5 mg/kg administered once monthly. Dose depends on actual body weight
OXLUMO® (lumasiran) Injection: 94.5 mg/0.5 mL in a single-dose vial	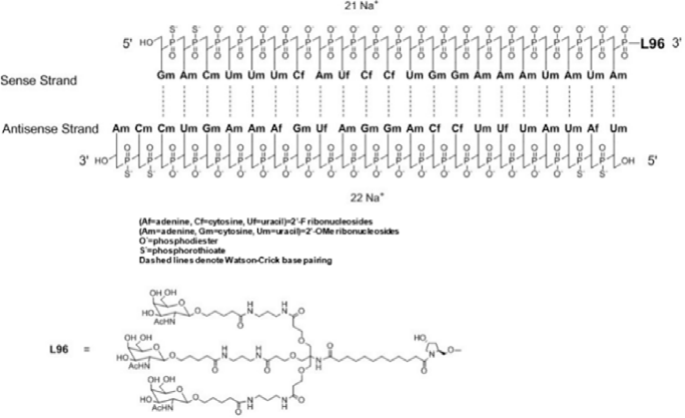	Loading dose followed by maintenance dose. Dose depends on actual body weight (loading and maintenance doses of 3–6 mg/kg)
AMVUTTRA® (vutrisiran) injection, for subcutaneous use	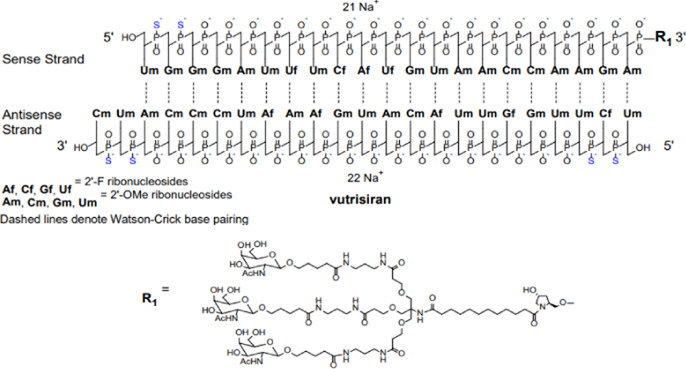	25 mg once every 3 months

Numerous NP systems have been employed to this end (Figure 5) and the exact contribution(s) of the NP features on the obtained response, especially their intrinsic activity to modulate immune response, are not always known. Nevertheless, NP design and engineering are gaining momentum to better fine-tune the cytokine response in a host. Large NPs with complex surface chemistry tend to induce stronger cytokine responses than those with smaller and simpler surface chemistry (Elsabahy and Wooley, 2013; Weiss et al., 2021). With peptide-based carriers, the resultant size and charge of the carrier can significantly influence the cytokine release (Tsai et al., 2020). The CPPs were generally found to be well tolerated, based on a range of *in vitro* and murine models ( Carter et al., 2013 ). The type of dendrimer used for nucleic acid delivery can also affect cytokine release, especially with their high density of regularly spaced charges (presenting a clearly artificial surface to the host). Cationic dendrimers have been reported to stimulate cytokine release more strongly than the anionic or neutral dendrimers (Moreira et al., 2023). Surface modification of dendrimers with certain functional groups or coatings can decrease cytokine release (Santos et al., 2020). With LNPs, special lipid compositions are being developed to minimize the immunogenicity (Tahtinen et al., 2022). The size of LNPs can also impact cytokine release, possibly due to differences in cellular uptake or intracellular processing. Hassett and others investigated the impact of a variety of physicochemical parameters on the immunogenicity of LNPs encapsulating mRNA expressing cytomegalovirus (CMV) pentamer, where CMV pentamer titers are known to manipulate cytokine production. The immunogenicity was assessed by antibody titers after prime (day 1) and booster (day 22) doses. The size of the LNP showed the best correlation with immunogenicity in the BALB/c mice model compared to other parameters. After the prime dose, anti-pentamer titers were increased with a particle size up to 105 nm, but subsequently reduced when the particle sizes increased. Following the boost, this correlation was found to be considerably more pronounced for LNPs with a particle size of less than 85 nm. (Hassett et al., 2021). The cytokine response to pDNA-incorporating cationic (DOTMA/Chol) liposomes were shown to be significant, but controllable to some degree by pre-treatment with gadolinium chloride (GdCl_3_) (Sakurai et al., 2002). Cationic polymeric carriers, analogues to dendrimers, can also elicit significant inflammatory cytokines and care is needed in polymer design to reduce reactive species (Beyerle et al., 2010), beyond simple PEGylation. Low MW PEIs (<2 kDa), either in native form or modified with various lipids, seems to be relatively free from cytokine elicitation (Meenakshi Sundaram et al., 2022). With a high MW (22 kDa) PEIs tested in a murine model, blood cytokine levels were elevated to between ∼4 and ∼10^3^ pg/mL with different batches of polymers, indicating the importance of a reproducible manufacturing process for the NP materials (Bonnet et al., 2008).

**FIGURE 6:**
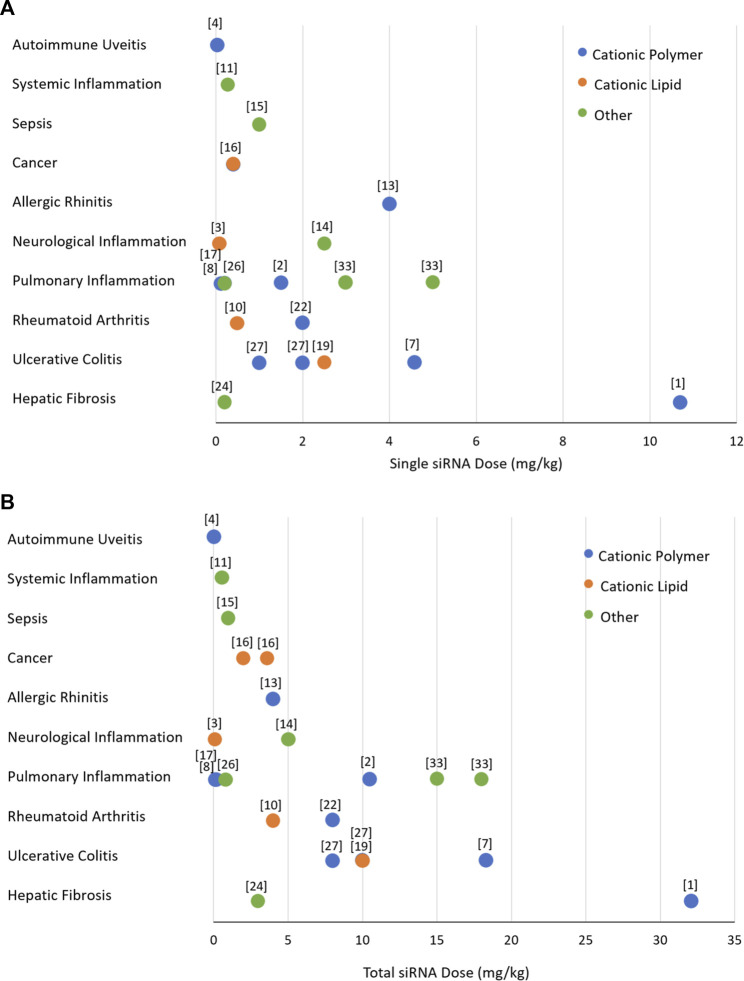
The doses of siRNA treatments used in preclinical models of inflammatory diseases. **(A)** The dose of individual siRNA injections. **(B)** Total dose injected based on the individual injection dose and the numbers of injections undertaken. The data is from studies summarized in Figure 5, excluding *in vitro*/cell culture studies. The numbers refer to the study references shown in the legend of Figure 5. The nature of delivery system used was classidied as polymeric (blue), lipid (orange) and others (green).

A close inspection of the reported RNAi studies in cytokine disorders indicated a significant variation in the administered dose (Figure 6). We expect a dose of ∼1 mg/kg in preclinical (animal) models to be reasonable for clinical translation to human patients. The doses used in most animal models are <5 mg/kg, with cationic polymers as well as lipids being effective at the lower dose of the spectrum. It is obviously not possible to directly assign the observed potencies (given by effective doses summarized in Figure 6) to the efficiency of the delivery system, since the target characteristics as well as the intrinsic potency of the siRNA to silence the intended target contribute to the overall potency. Head-to-head comparison are missing in most studies since the focus is to demonstrate the feasibility of a particulate nanoparticulate system in the intended indication. Dose response studies is a must in this regard, but admittedly as in our work, the feasibility of the therapeutic approach is commonly explored at a single “efficacious” dose rather than a dose range. It is noteworthy that the most efficacious systems (given by single and total doses administered) are polymeric chitosan and poly(lactide-co-glycolide), Transferrin-decorated and EpCAM (epithelial cell adhesion molecule)-decorated cationic liposomes, calcium phosphate, and arginine-functionalized AuNP (Cardoso et al., 2010; Chen et al., 2013; Frede et al., 2017; Jiang et al., 2018; Lian et al., 2019; Wu et al., 2021) indicating no preferential delivery system emerging among the effective systems. It appears that NPs of diverse compositions could be made to implement RNAi in preclinical models of cytokine disorders. Among these apparently more efficacious systems, the majority of the delivery systems were applied locally (Cardoso et al., 2010; Chen et al., 2013; Frede et al., 2017; Wu et al., 2021) that justified the relatively low dose, but some systemically (intravenous) as well (Jiang et al., 2018; Lian et al., 2019), making them promising for systems disorders.

## 5 Interpersonal differences in immune response

Inter-individual variations in human immune response, even among those considered as healthy, are widely accepted (as evident by differential responses in clinical studies), yet to which extent and for what reasons, not much is known. Using a systems-level analysis of 210 healthy twins between 8 and 82 years old, homeostatic cytokines such as IL-2 and IL-7 (crucial in T-cell proliferation) were found to be highly heritable whereas most signaling cytokines such as IL-6 and IL-21 were highly non-heritable (Brodin et al., 2015). This is in alignment with prior studies that demonstrated the low impact of heritability on variation in inter-individual T-cells and dendritic cell responses (Brodin and Davis, 2017). With age, genetic similarity in immune responses also decreased even in identical twins, with non-heritable influences contributing more to the variations (Brodin et al., 2015). These non-heritable impacts include environmental exposure (e.g., vaccines, infections, pollution, etc.), but can also extend to effects by pathogens, symbiotic microbiome and *de novo* mutations (Brodin and Davis, 2017). For example, a common environmental risk factor is cigarette smoke, which has been shown to correlate to higher autoantibodies as well as lower immunoglobulins and NK-cell functional activity (Brodin and Davis, 2017). Given the cross-talk between immune cell populations, a recent study has proposed a framework to view human immune systems in terms of immunotypes categorized based on key combinations of immune cell composition (Kaczorowski et al., 2017). The study found that variation in human immune systems is more continuous than discrete, and suggested that certain combinations of immune cell populations can predict functional responses to different stimuli (Kaczorowski et al., 2017). However, it should be noted that these analyses were limited to blood cell types, thus, an expansion of the measurements including serum cytokines and chemokines are necessary to validate and improve their predictive capacities (Kaczorowski et al., 2017).

The human immune responses to adenoviruses (AVs) were considered stereotypical, short-lasting and non-threatening, however, they manifest in a spectrum with diverse levels of length and severity (Atasheva and Shayakhmetov, 2022). Not surprisingly, patients with more severe diseases would exhibit more serious immune reactions after exposure to natural AV infections. Upon administration of AV therapies, IL-1 elevation was noted only 3 h after IV-administration, while early response cytokines such as IL-6, TNF-α, and IFN-γ and the CCL2 chemokine would show maximal levels at 6 h, with a return to baseline levels within 24 h. The presence of AV-specific antibodies can heighten the immune response by enabling viruses to gain entry into dendritic cells, which causes release of inflammatory cytokines (Atasheva and Shayakhmetov, 2022). It was the elevation of AV-specific antibodies that was suggested to potentially contribute to the lethal immune response in a patient after virus administration (Somanathan et al., 2020).

Regarding immune responses triggered by non-viral therapies, de Braganca and others have outlined both acute and long-term effects of the NP systems in the lung tissue (De Braganca, 2021). These effects can manifest locally such as edema and inflammation, but can also spread to organs beyond the pulmonary system. Immune side effects vary depending on the NP features such as size, geometry, stiffness, and hydrophobicity. Interestingly, higher toxicity is associated with smaller particles due to higher surface-volume ratio enabling more opportunities to interact with the immune system (De Braganca, 2021). Cationic NPs have been shown to trigger dendritic cell-activated release of pro-inflammatory factors such as IL-1β, IL-6, MCP-1, MIP-1α and TNF-α (Barillet et al., 2019), potentially due to interactions with the negative charges on the cell surface (De Braganca, 2021). Silica NPs also displayed immunotoxicity both *in vitro* and *in vivo;* genotoxicity and cytotoxicity were observed in RAW 264.7 cells upon exposure to such NPs at 200 μg/mL (Chen et al., 2018). Silica NP exposure also triggers size-independent injuries in mouse lung tissues as well as oxidative stress and cardiac inflammation in zebrafish embryos (Chen et al., 2018). Another reason behind adverse immunological side effects lies in the mechanism of NP administration. Commonly administered through inhalation, NPs can activate dendritic cells, whose pattern recognition receptors recognize the nucleic acid in the NPs as foreign (De Braganca, 2021). This triggers release of pro-inflammatory cytokines and chemokines and can cause cellular and organ damage (De Braganca, 2021). Non-viral gene therapeutics have been also shown to trigger immune responses in the ocular space. A recent review article by Ren and coworkers summarizes clinical findings that demonstrate even the widely considered immune-privileged ocular space is still subject to unwanted inflammatory effects elicited by retinal gene editing tools (Ren et al., 2022). Non-viral vectors such as chitosan showed inflammation and retinal damage while PCEP (poly(((cholesteryloxocarbonylamidoethyl) methylbis(ethylene) ammonium iodide) ethyl phosphate)) showed 15% retinal degeneration in rabbits (Ren et al., 2022). Similarly, lipoplexes carrying high amount of ribonucleoprotein showed toxicity in mice via subretinal delivery (Ren et al., 2022).

## 6 Conclusion and perspectives

The concerns related to cytokine release syndrome have been well appreciated in early gene therapy work with viral vectors, but this issue is also critical when it comes to non-viral systems. Although it has been possible to fine-tune this response and minimize it at times (Cron et al., 2023), it is still a concern with some non-viral NP systems. Non-viral NPs have lagged viral systems for clinical entry, and this is likely due to lower efficacy issues rather than the immunological reactions. Relatively minor measures can be taken to adjust the dose, administration route and especially to pre-screen patients to cytokine sensitivity, which will ultimately help with the host response. It may be possible to intervene with the cytokine responses very precisely, by relying on transient suppression of gene expression by using RNAi or more permanent measures such as CRISPR/Cas9 system (Ottaviano et al., 2022). Transient expression may be sufficient for controlling initial host response, which tends to subside if not life-threatening. A permanent stoppage of any cytokine response may give additional complications on the long term in managing host response to foreign invaders and/or other physiological and pathological events. RNAi can make it possible to intervene with a spectrum of cytokines in a convenient way, by using a mixture of specific siRNAs (early attempts have been made as noted in Figure 5). Considering the spectrum of attempts undertaken in several disease models, it is clear that there is a wide range of inflammatory and cytokine-associated diseases that will directly benefit from the RNAi approach. Where localized application is feasible (e.g., colitis with defined anatomical involvement), it may be safer to utilize nanoparticulate systems to locally deliver the cytokine interfering agents. Where systemic intervention might be required (e.g., sepsis), it is preferred to utilize “stealth” nanoparticulate systems that do not exasperate the disease any further. While PEG incorporation into NPs (or NP building blocks) has been the “go-to” approach for this end, this modification typically reduced the efficacy of NPs and needs to be implemented in a way that the dose administered are not compromised due to lower efficacy. The PEG itself, within the 2–10 kDa region, did not appear to induce proinflammatory cytokines with silica NP incubated primary murine macrophages and dendritic cells (Storjohann et al., 2023). However, other types of NPs and/or dose used were reported to significantly affect this response (Tehrani et al., 2022; Zamorina et al., 2023). Coating the surface of NPs or including additives that change the zeta-potential (from highly cationic surface to neutral surface, in our hands) could be a simpler measure to reduce inflammatory cytokine response (Meenakshi Sundaram et al., 2022). The exact role of NP features when attempting to modulate cytokine secretion needs to be better controlled. Clearly, the collective experience in various preclinical models indicated that the cytokine response is a significant issue with all of the NP delivery systems (Godinho et al., 2014). When RNAi is attempted to be implemented, the NP delivery system should not be viewed as a simple ‘passive’ carrier; it may elicit or help to reduce cytokine levels independently. It is likely that particular NPs in the spectrum of NPs used (Figure 5) will be more suitable for certain applications. Efforts to elucidate this relationship is worthwhile since it will critically impact the efficacy and the course of pharmacodynamic response in the investigated pathophysiology.

Using human PBMCs rather than animal models is important in assessing the cytokine response. We found cells lines such as Jurkat T-cells to be much more sensitive than the primary PBMC, which may lead to an erroneous assessment of the cytokine response if used routinely. Individual variations in cytokine response are obviously better modeled by the use of PBMC in culture (Figure 3). It will be important to tackle this with a representative sampling of PBMCs to estimate the true potential of delivery systems to induce inflammatory cytokines. Ultimately, this will save much effort to select minimally reactive delivery systems, where the clinical safety is utmost important.

While a great deal of efforts have been placed on minimizing cytokine response, it may be useful in certain indications to utilize NPs that can elicit a specific cytokine response. As eluted in experimental cancer applications above, induction of cytokine response can aid in therapeutic effects for certain kinds of cancers, as in the case of sensitizing malignant cells to chemotherapy. In that way, better understanding of structural features of the NPs that can elicit a strong and/or selective cytokine response will help to design novel systems for therapy. Induction of a certain cytokine cocktail has the potential to alter the physiology of local immune cells so that the effects might be long lasting and more potent upon such an ‘engineered’ cytokine response. For example, with lipid-substituted PEI polymers, we have observed that cytotoxicity, hemocompatibility (i.e., RBC lysis potency) and inflammatory cytokine (TNF-α, IL-6 and IFN-γ) secretion was differentially dependent on the nature of the lipid substituted on the PEI (Meenakshi Sundaram et al., 2022). Hence it may be possible to fine-tune these properties independent of each other. While these cytokines are lumped as inflammatory cytokines, we also saw differential upregulation of the cytokines in response to non-viral carrier exposure, in that one or more of this class of cytokines could be selectively stimulated, so that it may be possible to fine tune the response among a physiologically similar group of cytokines as a function of carrier features. Individual variations in cytokine response and its determinants constitute another aspect of NP technology that will require more attention to develop personalized therapies and optimize NPs for gene delivery, so that research efforts need to continue to shed more light on this important issue.
